# *Trypanosoma cruzi* Genomic Variability: Array Comparative Genomic Hybridization Analysis of Clone and Parental Strain

**DOI:** 10.3389/fcimb.2022.760830

**Published:** 2022-03-25

**Authors:** Danielle Rodrigues Cortez, Fabio Mitsuo Lima, João Luís Reis-Cunha, Daniella Castanheira Bartholomeu, Rolando Andre Rios Villacis, Silvia Regina Rogatto, André Guilherme Costa-Martins, Fernanda Sycko Marchiano, Rafaela Andrade do Carmo, Jose Franco da Silveira, Marjorie Mendes Marini

**Affiliations:** ^1^Departamento de Microbiologia, Imunologia e Parasitologia, Escola Paulista de Medicina, Universidade Federal de São Paulo, São Paulo, Brazil; ^2^Centro Universitário São Camilo, Biomedicina, São Paulo, Brazil; ^3^Departamento de Parasitologia, Instituto de Ciências Biológicas, Universidade Federal de Minas Gerais, Belo Horizonte, Brazil; ^4^Departamento de Genética e Morfologia, Instituto de Biologia, Universidade de Brasilia, Brasilia, Brazil; ^5^Department of Clinical Genetics, Institute of Regional Health Research, University of Southern Denmark, Vejle, Denmark; ^6^Department of Clinical and Toxicological Analyses, Faculdade de Ciências Farmacêuticas, Universidade de São Paulo, São Paulo, Brazil

**Keywords:** *Trypanosoma cruzi*, intrastrain variability, parental strain and clone, karyotyping, array comparative genomic hybridization, gene copy number variation, chromosome rearrangement, aneuploidy

## Abstract

*Trypanosoma cruzi*, the etiological agent of Chagas disease, exhibits extensive inter- and intrastrain genetic diversity. As we have previously described, there are some genetic differences between the parental G strain and its clone D11, which was isolated by the limiting dilution method and infection of cultured mammalian cells. Electrophoretic karyotyping and Southern blot hybridization of chromosomal bands with specific markers revealed chromosome length polymorphisms of small size with additional chromosomal bands in clone D11 and the maintenance of large syntenic groups. Both G strain and clone D11 belong to the *T. cruzi* lineage TcI. Here, we designed intraspecific array-based comparative genomic hybridization (aCGH) to identify chromosomal regions harboring copy-number variations between clone D11 and the G strain. DNA losses were more extensive than DNA gains in clone D11. Most alterations were flanked by repeated sequences from multigene families that could be involved in the duplication and deletion events. Several rearrangements were detected by chromoblot hybridization and confirmed by aCGH. We have integrated the information of genomic sequence data obtained by aCGH to the electrophoretic karyotype, allowing the reconstruction of possible recombination events that could have generated the karyotype of clone D11. These rearrangements may be explained by unequal crossing over between sister or homologous chromatids mediated by flanking repeated sequences and unequal homologous recombination *via* break-induced replication. The genomic changes detected by aCGH suggest the presence of a dynamic genome that responds to environmental stress by varying the number of gene copies and generating segmental aneuploidy.

## Introduction

Chagas disease is a neglected tropical disease with a global prevalence of 6-7 million infected people that causes more than 10,000 deaths every year (Coura and Viñas, 2010; WHO, 2021). *Trypanosoma cruzi*, the etiological agent of Chagas disease, is a flagellate protozoan of the order Trypanosomatida, which comprises species that diverged from the main eukaryotic lineage early in their evolution. The population structure of *T. cruzi* has been shown to be predominantly clonal, but there is extensive evidence of genetic exchange and natural hybridization, including meiotic sex, between strains (Tibayrenc et al., 1990; Westenberger et al., 2005; de Freitas et al., 2006; Tibayrenc and Ayala, 2013; Berry et al., 2019; Schwabl et al., 2019). Natural populations of *T. cruzi* exhibit a broad spectrum of genotypic and phenotypic traits and have been grouped into six discrete typing units (DTUs) known as lineages TcI-VI (Ackermann et al., 2012; Zingales et al., 2012).

Whole genomic analyses by next-generation sequencing (NGS) technologies have shown extensive genomic variability and aneuploidy among isolates from different *T. cruzi* lineages (Franzén et al., 2011; Ackermann et al., 2012; Reis-Cunha et al., 2015; Bradwell et al., 2018; Berná et al., 2018; Callejas-Hernández et al., 2018) and within isolates of the same lineage (Reis-Cunha et al., 2018). Minning et al. (2011) compared 17 *T. cruzi* strains using array comparative genomic hybridization (aCGH). They observed extensive, widespread gene copy number variation (CNV) among the isolates, suggesting that the parasite is tolerant to CNVs and even aneuploidy. Reis-Cunha et al. (2015) identified notably few aneuploidy events in isolates from the TcI lineage, while isolates from TcII and TcIII lineages had a large number of chromosomal expansions.

Molecular karyotyping analysis by pulsed field gel electrophoresis (PFGE) and hybridization with chromosome-specific markers demonstrated that *T. cruzi* exhibits extensive inter- and intrastrain karyotypic heterogeneities, suggesting gross chromosomal rearrangements (Henriksson et al., 1996; Porcile et al., 2003; Souza et al., 2011; Lima et al., 2013). McDaniel and Dvorak (1993) reported the presence of intrastrain chromosome rearrangements in naturally occurring variants of the Y-02 stock of the *T. cruzi* Y strain. In a previous report, we described differences between the karyotypes of the parental G strain and its clone D11, a clone isolated by the limiting dilution method and by infecting cultured mammalian cells *in vitro* (Lima et al., 2013). The karyotype of clone D11 differs in number and size of chromosomal bands from the karyotype of the G strain, and chromosomal rearrangements were detected by Southern blot hybridizations with chromosome-specific markers. In most cases the chromosome length polymorphisms were small, resulting in the appearance of additional chromosomal bands in clone D11. Despite the karyotypic alterations, large syntenic groups were conserved, suggesting that core genomic regions are essential for development of the parasite (Lima et al., 2013).

The occurrence of intrastrain genetic variability in *T. cruzi* has been investigated by different approaches. Clones derived from the same parental strain can differ from each other in antigenic composition (Brenière et al., 1991); growth and virulence (Postan et al., 1983; Campos and Andrade, 1996); and zymodeme (Dvorak et al., 1980; Goldberg and Silva Pereira, 1983) and kDNA profiles (Morel et al., 1980). Recently, it has been demonstrated that isogenic clonal cell lines of *T. cruzi* have varying phenotypes, including expression of surface protein and fitness‐determining traits (Seco-Hidalgo et al., 2015). Clones CLB and CL-14 of the CL strain display contrasting virulence phenotypes in a murine model of infection. Comparative transcriptome analysis indicates that the avirulent phenotype of CL-14 may be related to reduced or delayed expression of surface protein genes (Belew et al., 2017).

Although several reports have shown the presence of clonal and intrastrain karyotypic differences and CNVs in *T. cruzi*, the clonal heterogeneity in this parasite remains to be explained. In this study, we carried out a more in-depth comparative genomic analysis of the parental G strain and clone D11. By combining aCGH, PFGE karyotyping and hybridization with specific chromosome markers, we identified gene and chromosome copy-number differences between the G strain and clone D11, which could be a source of genomic variation. Our results may help to elucidate *T. cruzi* response to stress and the mechanism responsible for genome plasticity in this parasite.

## Methods

### Parasites and DNA Extraction

Clone CL Brener (CLB) (*T. cruzi* Discrete Typing Unit (DTU) VI- lineage TcVI) (Zingales et al., 1997; Zingales et al., 2009; Zingales et al., 2012) and the G strain (*T. cruzi* DTU I - lineageTcI) (Yoshida, 1983; Briones et al., 1999; Zingales et al., 2009; Zingales et al., 2012) in axenic cultures at 28°C in liver-infusion tryptose medium (LIT) containing 10% fetal calf serum. Clone D11, which also belongs to the DTU I - lineageTcI (Lima et al., 2013), was isolated from the G strain by Santori (1991) using the protocol described by Lima and Villalta (1989). Log-phase epimastigotes were washed in phosphate buffered saline (PBS) and collected by centrifugation. Genomic DNA extraction was performed with the DNeasy Blood & Tissue (Qiagen) kit using 5x10^7^ cells/mL according to the manufacturer’s instructions.

### Separation of *T. cruzi* chromosomal bands by pulsed-field gel electrophoresis (PFGE) and Southern blot analysis

Approximately 1 x 10^7^ epimastigotes in exponential phase were washed with PBS and collected by centrifugation; an equal volume of cell suspension and 2% low-melting point agarose were mixed as previously described (Souza et al., 2011). The chromosomes were separated by pulsed-field gel electrophoresis (PFGE) and hybridized with the indicated markers labelled with (Zingales et al., 1997)P as previously described (Cano et al., 1995; Souza et al., 2011). The genes used as probes are indicated in the figure legends.

### aCGH Design, Hybridization and Analysis

The 8x60K array was designed and produced by Agilent (Agilent Technologies, Santa Clara, CA,US) based on the *T. cruzi* clone CLB genome, release 6, available in TriTrypDB (http://tritrypdb.org/tritrypdb/; see **Supplementary Table S1**). CLB genomic contigs were assembled into 41 *in silico* chromosome (TcChr) pairs varying in size from 78 kb to 2.3 Mb (Weatherly et al., 2009). Because of the hybrid origin of CLB, the genome of this strain is composed of two parental haplotypes, designated Esmeraldo-like (S) and non-Esmeraldo-like (P) (El-Sayed et al., 2005). Two groups of probes totaling 48,787 probes covering all the *in silico* chromosomes were selected. The first group of probes covered all the chromosomes, including coding and non-coding regions, with an average of one probe every 1.2 kb to give a total of 44,860 probes. To design the second group, we selected 422 specific chromosome markers (i.e., single copy genes present only in the homologous chromosomes). In order to obtain a higher coverage with these markers, probe density was increased to one probe every 120 bp.

Raw data was first normalized using Feature Extraction software (Agilent technologies). Agilent Genomic Workbench Standard Edition 6.5 was then used to perform CNV interval detection. The QC metrics motif of Workbench 6.5 ensured adequate quality control of the hybridization data. In our study, an array signal which met the requirements if intensity value >50 and signal-to-noise ratio >25 was included in the analysis. The Aberration Detection Method 2 algorithm was used with a threshold of 6 and bin size of 10 to identify genomic variation. The aCGH scanning step for data acquisition and extraction was carried out by the Agilent Feature Extraction Software using the default of 0.25 log2 ratio (log*2* ratio *>* 0.25 for gains and log*2* ratio *< −*0.25 for losses). Additionally, we applied a relatively stringent post-analysis filter to ignore small, spurious or low-quality alterations (Wang et al., 2015). We used as defining criteria of copy number alterations the presence of at least four consecutive probes altered in the region, minimum average absolute log2 ratio 0.5, Log2 ratios > 0.5 and < –0.5 as the threshold for gain and loss, respectively.

The raw aCGH data and sequence of probes involved in the aCGH design for Trypanosoma cruzi have been deposited into the GenBank GEO database (GSE197870). The GenBank GEO accession number (GSE197870) (http://www.ncbi.nlm.nih.gov/geo/).

### Quantitative Real Time PCR Validation

qPCR was used to validate the aCGH data. Primer amplification efficiency for each gene was determined. Specific sequences of the reference and target genes were cloned into pGEM-T easy vector (Invitrogen). Known amounts of genomic DNA (from clones D11 and CLB and G strain) and recombinant plasmids containing the sequences of interest were incubated with 10 µL SYBR Green-Based Detection (Applied Biosystems), sense and antisense primers and water to a total reaction volume of 20 µL. The reaction mixture was distributed into 0.2 mL tubes and subjected to 40 cycles of amplification in the Rotor-Gene^®^ Q PCR cycler (Qiagen) according to the manufacturer’s instructions. The qPCR program was set as follows: initial denaturation at 95°C for 5 min, 40 cycles of denaturation at 95°C for 15 s, annealing and extension at 60°C for 60 s. The results were analyzed with Rotor-Gene 6000 v1.7 software (Qiagen). A standard curve was constructed for each target gene. Data obtained by amplification with genomic DNA samples could be compared as the amount of genomic DNA was the same for the three *T. cruzi* isolates. To estimate the copy numbers of each target gene in the three isolates, data were normalized separately using the genome size of each *T. cruzi* isolate (Souza et al., 2011). All experiments were performed in triplicate.

### Content of Amplified and Deleted Gene Regions and Chromosomal Distribution

To estimate gene content in amplified and deleted regions in clone D11 in relation to that of Esmeraldo-like and Non-Esmeraldo-like haplotypes, the coordinates of the regions that varied in copy numbers were crossed with the gene coordinates in CLB General Feature Format (GFF) files, version 9, downloaded from TriTrypdb (http://tritrypdb.org/tritrypdb/) using BEDTools intersect v2.23 **(** Quinlan and Hall, 2010**)**. Initially, all genes whose coordinates were inside the deleted or amplified regions were counted and classified as follows: 1- Multigene Families (MGFs.) if they were annotated as Trans-sialidase (TS), Mucins (TcMUC), Mucin Associated Surface Proteins (MASP), Retrotransposon Hotspot Protein (RHS), surface glycoprotein gp63 (GP63) or Dispersed Gene Family 1 (DGF-1); 2- Hypothetical if they were annotated as Hypothetical Proteins; and 3- Others if they did not meet any of the above criteria. To compare the MGF content of the amplified or deleted regions, the proportion of each multigene family was determined.

Visualization of the D11 amplified or deleted regions with the Esmeraldo-like or Non-Esmeraldo-like *in silico* chromosomal sequences was performed using Perl scripts and the R libraries grid (https://stat.ethz.ch/R-manual/R-devel/library/grid/html/00Index.html), ade4 (https://cran.r-project.org/web/packages/ade4/index.html) and genoPlotR (http://genoplotr.r-forge.r-project.org/). The length of the chromosomes was drawn according to the coordinates in the Esmeraldo-like and Non-Esmeraldo-like genome FASTA files, version 9, downloaded from TriTrypdb (http://tritrypdb.org/tritrypdb). Each gene was drawn in its position and strand based on the GFF file, where grey boxes correspond to housekeeping and hypothetical genes and cyan boxes denote MGF genes. The green and red boxes correspond to deleted and duplicated regions, respectively.

The whole genome sequence (WGS) of *T. cruzi* isolate Dm28c-version 2018 (lineage TcI) was downloaded from http://tritrypdb.org/tritrypdb. Whole genome alignments between CLB chromosomes (TcChr S - Esmeraldo-like haplotype and TcChr P - non-Esmeraldo-like haplotype) and Dm28c contigs were performed using the blastn algorithm and implemented with big_blast.pl script from the Sanger Institute. The annotation and graphical output of chromosome-specific markers were obtained using the Artemis Comparison Tool (ACT) (Carver et al., 2005) (http://www.sanger.ac.uk/resources/software/act).

## Results

### Design of the Array and Experiments

Clone D11 and its parental G strain belong to the DTU I – lineage TcI of *T. cruzi* (Briones et al., 1999; Zingales et al., 2009; Zingales et al., 2012; Lima et al., 2013). The genome of clone CL Brener (CLB) of lineage TcVI (Zingales et al., 1997; El-Sayed et al., 2005; Zingales et al., 2009; Zingales et al., 2012) was used as the DNA reference in the microarray design because it was the only *T. cruzi* genome assembled into chromosomes (Weatherly et al., 2009). Genomic sequences of clone CLB, the reference strain of the *T. cruzi* genome, have been previously assembled into chromosome-sized scaffolds that allowed the array to be designed. CLB genomic contigs were assembled into 41 *in silico* chromosome (TcChr) pairs that varied in size from 78 kb to 2.3 Mb (El-Sayed et al., 2005). Because of the hybrid nature of CLB, the genome of this strain is composed of two parental haplotypes designated Esmeraldo-like (S) and non-Esmeraldo-like (P). Nearly 50% of the *T. cruzi* genome is comprised of repetitive sequences, such as multigene families (MGFs), retrotransposons and micro- and mini-satellites (El-Sayed et al., 2005). The final assembly of chromosomes was hampered by its hybrid nature and the many repetitive sequences, which means that a considerable number of unassigned contigs are found in the *T. cruzi* database. Although the G strain (TcI) and clone CLB (TcVI) belong to two distantly related lineages, there is evidence that large syntenic regions are conserved among the different *T. cruzi* lineages (Souza et al., 2011). This has recently been confirmed by Next Generation Sequencing (NGS) procedures (Berná et al., 2018; Callejas-Hernández et al., 2018). To compare sequence divergence between the genomes of isolates from TcVI and TcI lineages, we evaluated the homology of individual chromosomes of CLB (TcVI) with their counterparts in Dm28c (TcI) whose assembly in large contigs was recently published (Berná et al., 2018). We confirmed gene sequence identity between CLB and Dm28c, and the conservation of gene order (synteny) in the same relative positions in the chromosomes of these isolates (**Supplementary Figure S1**). The differences found between CLB and Dm28c are usually due to variation in the number of copies of MGFs and genes encoding small RNAs (snRNA, sonRNA, tRNA). These results support our aCGH analysis comparing the genomes of the G strain and clone D11 with that of CLB.

The DNA content of the G strain [genome size=89.8 Mb (Lima et al., 2013)] and clone D11 were assumed to be the reference and test DNAs, respectively. This assumption has taken into account that clone D11 was isolated from strain G. Therefore, alterations related to DNA gains in the G strain that did not occur in clone D11 were assumed to be DNA losses in clone D11; conversely, DNA losses in the G strain were assumed to represent DNA gains in clone D11. We assumed that each clone D11 chromosome is homologous to a CLB chromosome (TcChr). For example, TcChr12 chromosome markers identified DNA gain and loss in the homologous of clone D11, which will be referred to here as a D11 chromosome homologous to TcChr12 of CLB.

### Detailed Features of Chromosomal Alterations in Clone D11

aCGH can be used to identify losses and gains, including imbalances associated with apparently balanced translocation, i.e., duplication followed by translocation. However, it is unable to detect balanced chromosomal rearrangements, e.g., inversions and balanced reciprocal translocations. The bulk of the detailed information on chromosomal alterations found in clone D11 is presented in **Supplementary Table S2**, which includes the *in silico* CLB chromosomes to which they were mapped, the genomic location (the beginning and end of the alteration), the size and type of alteration (DNA loss or gain) and the number of probes for each alteration. Of the 418 chromosomal imbalances identified in clone D11, 144 (34.45%) were classified as DNA gain/amplification, and 274 (65.55%) as DNA loss/deletion. It is interesting to note that the parameters used in our analysis to identify DNA alterations were not equally stringent for defining gain and loss. A more restrictive threshold was used to define DNA gain than DNA loss. To increase the stringency and selection of non-random alterations, we have used at least 4 consecutive altered probes. However, we can not rule out that the high proportion of DNA loss over DNA gain in clone D11 may also be due to a more stringent threshold for defining DNA gain. The average size of chromosomal alterations was 23 kb, the smallest being 1.5 kb, and the largest 405 kb (**Supplementary Figure S2**). Most chromosomal alterations (89.71%) were smaller than 50 kb, and only six were >200 kb.

Chromosomal alterations identified in clone D11 were mapped on both haplotypes (S, Esmeraldo-like and P, Non-Esmeraldo-like) of clone CLB. The proportion of chromosome alterations (loss or gain) in each chromosome was estimated based on the length of CLB chromosomes (Weatherly et al., 2009) (**Supplementary Figure S3**). CNVs were distributed in a heterogeneous manner throughout the D11 genome and their proportion to a given chromosome varied from 1.1% to 89.3%. Deletion and duplication proportions appear to vary among D11 chromosomes; notably, losses (65.55%) prevailed over gains (34.45%). The majority of D11 chromosomes exhibited some alteration, except the chromosome that shares homology with TcChr4 of CLB. Eighteen chromosomes had only DNA losses whereas in four chromosomes only DNA gain was observed. There was no apparent correlation between chromosomal length and number of chromosomal alterations. The proportion of chromosomal alterations in D11 was very similar to that in the haplotypes of CLB.

**Figure 1:**
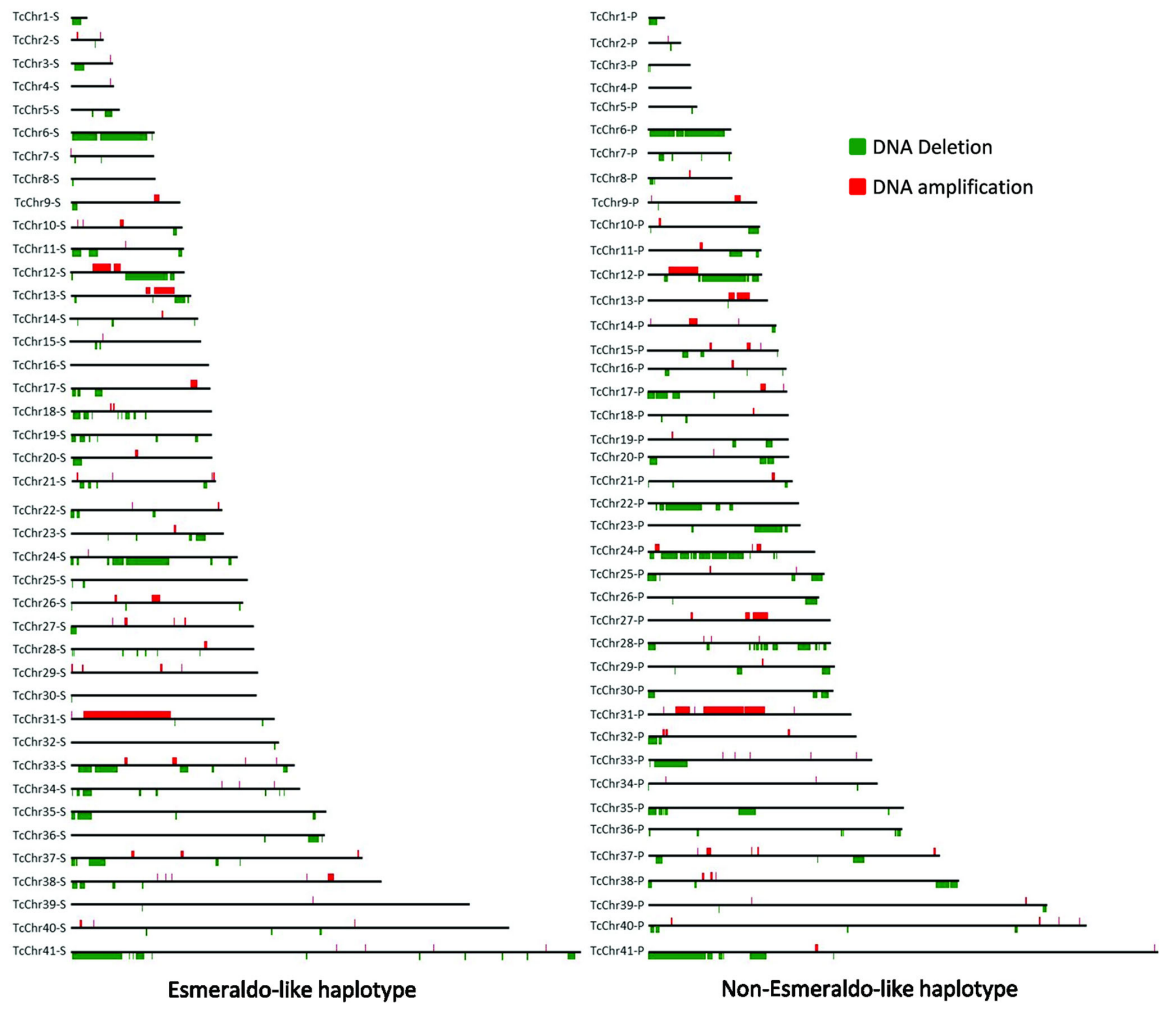
Distribution of CNVs across *T. cruzi* chromosomes. Chromosomal alterations identified in clone D11 were mapped on both haplotypes (S, Esmeraldo-like and P, Non-Esmeraldo-like) on each *in silico* chromosome of the CLB reference genome (Weatherly et al., 2009). *In silico* chromosomes of CLB are numbered 1 to 41, from the smallest, TcChr1 (0.77 Mb), to the largest, TcChr41 (2.37 Mb). Amplification and deletion regions are represented in red and green, respectively. Chromosomes and recombination events are represented to scale.

The distribution of CNVs in the chromosomes is shown in **Figure 1**. The bars are scaled according to chromosome size and show the predominance of DNA loss over DNA gain across the chromosomes. We selected several examples of chromosomes with different CNV profiles for a more comprehensive analysis (**Supplementary Figure S4**). For instance, a profile with a few loss/gain events was detected in the D11 chromosomes homologous to TcChr37, TcChr39 and TcChr8 of CLB.

Chromosomes TcChr4 and TcChr37 are part of a single chromosome and syntenic group that is highly conserved among different *T. cruzi* lineages (Souza et al., 2011). Another syntenic group was identified in TcChr39 (Souza et al., 2011). The syntenic groups (TcChr4+TcChr37) and TcChr39 are much larger than would be expected if rearrangements occurred randomly, suggesting that they have undergone positive selection (Souza et al., 2011). The aCGH analysis agreed with this hypothesis since chromosomes TcChr39 and (TcChr4+TcChr37) exhibited very few CNVs. In a number of cases, a large part of the chromosome had changed. The D11 chromosome homologous to TcChr6 lost a large region comprising 89% of the entire chromosome whereas the D11 chromosome homologous to TcChr31 showed duplication of a segment covering 41% of the chromosome. A mixed CNV pattern was found in the D11 chromosome that shares homology with TcChr12. This chromosome has a large segment with loss and gain regions that correspond to 80% of the chromosome. Several D11 chromosomes showed different hybridization patterns to those of the haplotypes of CLB. For example, the D11 chromosome homologous to TcChr22-P had a large DNA loss region (32% of the length of the chromosome) that was absent in the TcChr22-S haplotype. Another D11 chromosome had a deletion that corresponded to 36% and 57% of the Tchr24-S and Tchr24-P haplotypes, respectively. Notably, some D11 chromosomes are highly conserved with changes in less than 2% of their lengths, e.g., the D11 chromosomes homologous to TcChr39 and TcChr37. Alterations in TcChr39 correspond to only 0.76% of the whole chromosome (approximately 1.85 Mb). These results agree with previous works that showed a remarkable conservation of TcChr39 and TcChr37 in different *T. cruzi* lineages (Santos et al., 1999; Souza et al., 2011; Lima et al., 2013).

### Gene Content of Chromosomal Alterations in Clone D11

**Figure 2:**
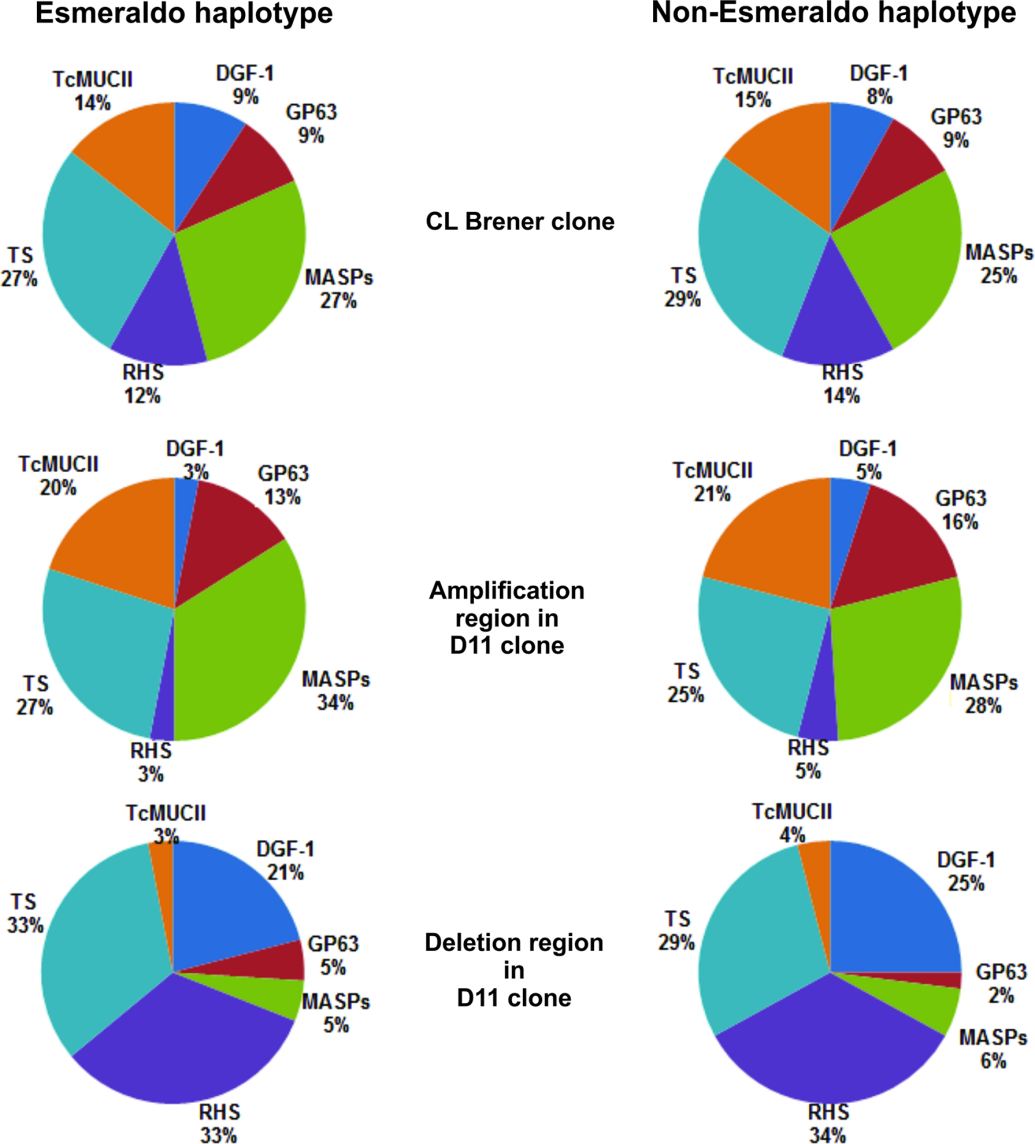
Distribution of multigene families in the chromosomal alterations of clone D11. The distribution of multigene families in amplification and deletion regions was compared with the gene content of both haplotypes (Esmeraldo and non-Esmeraldo) of the CLB reference genome. The CLB gene contents were compared with those in the amplification and deletion regions of clone D11: TS, Trans-Sialidase; TcMUCII – *T. cruzi* Mucin II; DGF-1, Dispersed Gene Family 1; Gp63; MASP – Mucin Associated Surface Protein; RHS, Retrotransposon Hot Spot.

To determine the gene content of the chromosomal alterations, we analyzed 418 alterations in clone D11 using gene annotation (**Supplementary Table S3**). We identified 2,199 genes involved in DNA losses: 1,032 genes (of which 18.99% were pseudogenes) in Esmeraldo haplotypes and 1,167 genes (of which 24.67% were pseudogenes) in non-Esmeraldo haplotypes. A relatively small number of genes were identified in the amplified regions: 353 genes (of which 13.03% were pseudogenes) in Esmeraldo haplotytpes and 328 genes (of which 17.68% were pseudogenes) in non-Esmeraldo haplotypes ((**Supplementary Table S3**). The annotated genes were distributed into three groups: a 1^st^ group, consisting of MGFs encoding surface proteins, such as trans-sialidases, MASPs, TcMUC (mucins), GP63 and DGF1 (dispersed gene family 1), and nuclear proteins coded by the RHS gene family (retrotransposon hot spot protein); a 2^nd^ group, consisting of genes encoding hypothetical proteins without an assigned function; and a 3^rd^ group consisting of other genes that do not meet any of the above criteria, such as replication protein genes, kinases and phosphatases (**Supplementary Figure S5**). There was an increase in the proportion of MGFs in clone D11 in relation to the G strain. The gene content of MGFs in DNA gain regions clearly differs from those with DNA loss (**Figure 2**). We observed an increase in MASP, mucins (TcMucII) and Gp63 content in the amplification regions of clone D11 whereas in the deletion regions there was a decrease in the content of these genes and an increase in RHS and DGF1.

**Figure 3:**
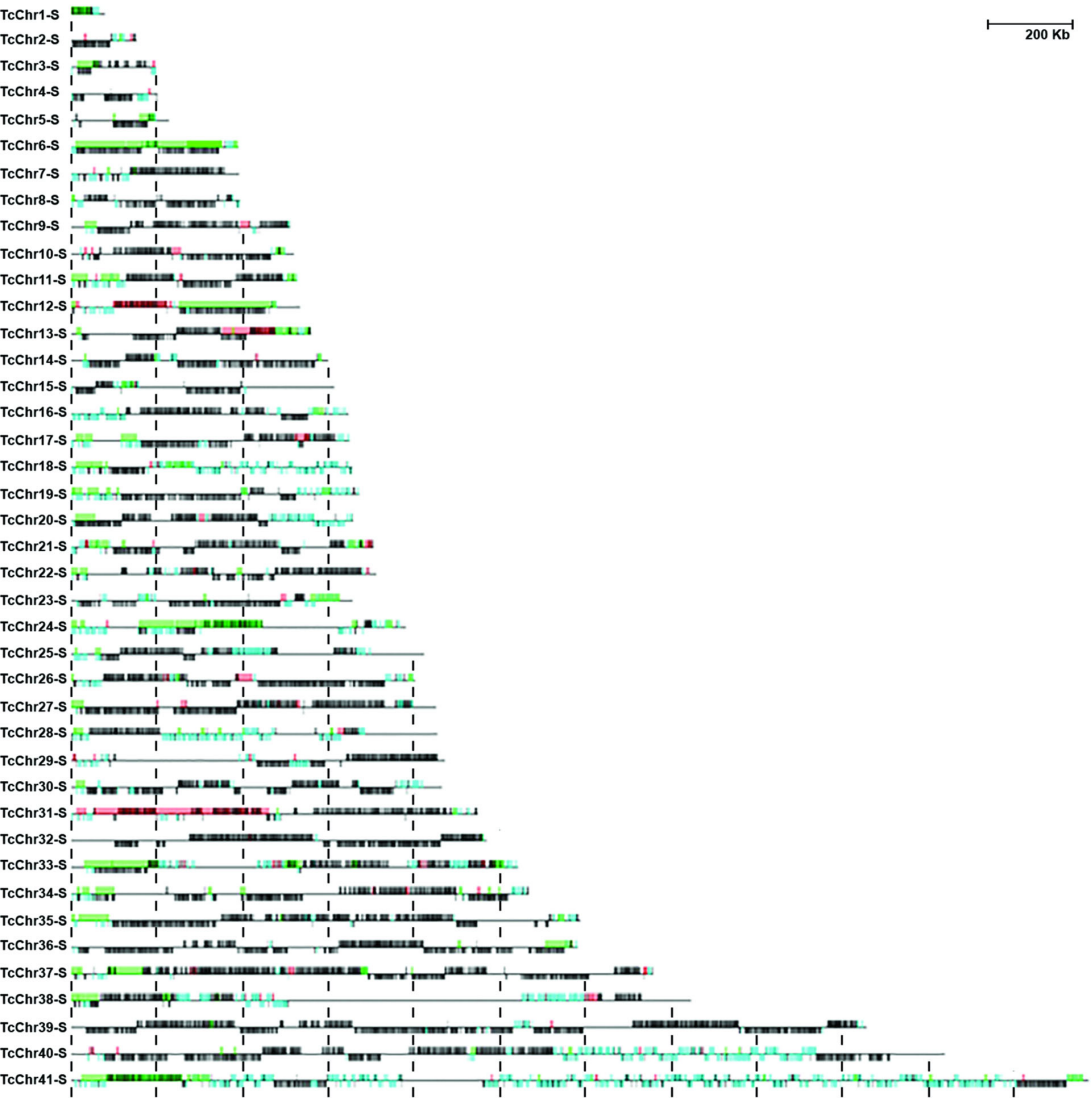
Schematic representation of *in silico* chromosomes of Esmeraldo-like haplotype showing the gene annotation and chromosomal alterations detected by aCGH in clone D11. Annotation of multigene families in CLB is shown in light blue, and the other genes in black. The CNVs detected by aCGH in clone D11 are shown on the *in silico* CLB chromosomes. DNA amplification and deletion are shown in red and green, respectively. Chromosomes and recombination events are shown to scale (Bar=200 kb).

We speculated about a possible association between the presence of MGFs and chromosomal alterations and therefore mapped the alterations (deletions in green and amplifications in red) detected in the D11 clone with the gene annotation (MGFs in light blue and other genes in black*)* in the *in silico* S and P haplotypes of CLB chromosomes (**Figure 3** and **Supplementary Figure S6**). However, we found that the CNVs were unevenly distributed along the chromosomes, and there seems to be no connection between the abundance or size of multigene family clusters (represented in light blue) and the number and size of chromosomal alterations found in clone D11. For instance, the presence of many members of MGFs throughout chromosomes TcChr18 and TcChr41 was in striking contrast to the small number of alterations in these chromosomes. On the other hand, chromosomes TcCrh6 and TcChr31 harbor a small number of genes belonging to MGFs, but many chromosomal alterations could be observed. In agreement with a previous report, the pattern of chromosomal alterations flanked by MGFs was observed (Minning et al., 2011). The authors reported that in several strains of *T. cruzi*, CNVs were particularly frequent in gene family-rich regions containing mucins and trans-sialidases.

### Gross Chromosomal Rearrangements in Clone D11

The electrophoretic karyotypes of clone D11 and the G strain differ in number and size of chromosomes (Lima et al., 2013). To facilitate understanding and comparison of CNVs between the G strain and clone D11, the chromosomal bands of these isolates were separated by PFGE using the electrophoretic conditions established by Lima et al., 2013 (Lima et al., 2013) (**Supplementary Figure S7**). Chromosome size differences between clone D11 and G strain ranged from approximately 40 to 340 kb, suggesting small chromosome rearrangements in clone D11. In this study we carried out Southern blot hybridizations using chromosome-specific markers within or near the chromosomal regions showing alterations detected by aCGH. We also looked for possible balanced recombination events that could not be identified by aCGH such as translocations.

**Figure 4:**
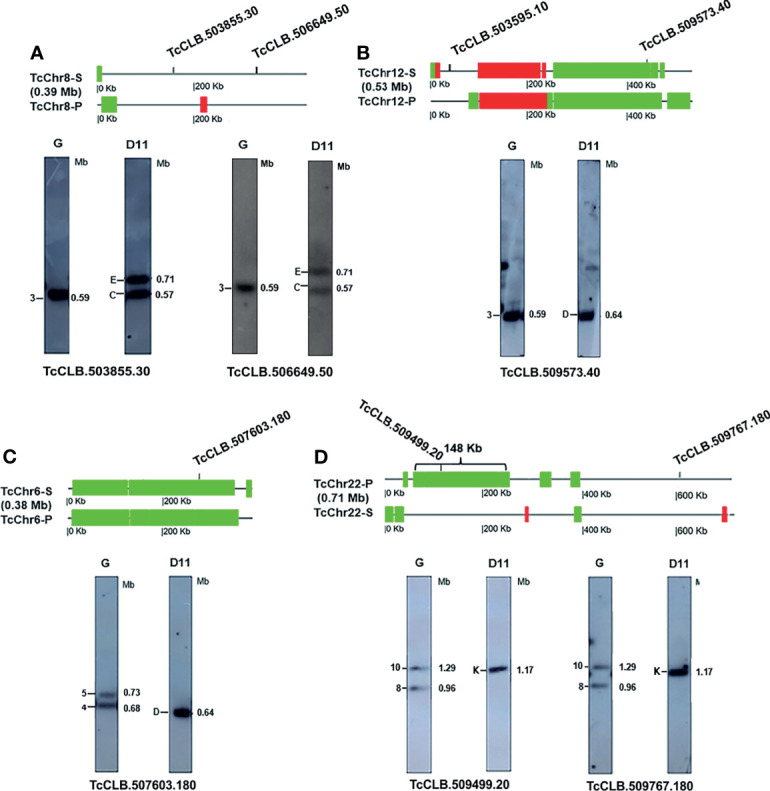
Gross chromosomal rearrangements in clone D11 detected by aCGH and chromoblot hybridization. At the top of each panel there is a schematic representation of *in silico* CLB chromosomes showing the CNVs detected in clone D11 by aCGH. DNA amplification and deletion are shown in red and green, respectively. *In silico* chromosomes and CNVs are shown to scale. Below this is the Southern blot hybridization of chromosomal bands of the G strain and clone D11 separated by PFGE with chromosome-specific markers labeled with (Zingales et al., 1997)P (see the raw image in **Supplementary Figure S8**). The number and size (Mb) of chromosomal bands are indicated. The position of the chromosome-specific marker is indicated in the CLB chromosomes. The *in silico* chromosomes and markers are: **(A)** TcChr8, TcCLB.510337.30 - adenylosuccinate lyase; TcCLB.506649.50 - cytochrome c oxidase assembly factor **(B)** TcChr12, TcCLB.509573.40 - hypothetical protein; TcCLB.503595.10 - hypothetical protein **(C)** TcChr6; TcCLB.507603.180 - ubiquitin hydrolase 6 **(D)** TcChr22; TcCLB.509499.20 - beta propeller protein 1 BPI; T; TcCLB.509767.180 - exosome component CSL4.

Representative examples of rearrangements in clone D11 are shown in **Figure 4** (see raw images in **Supplementary Figures S8A-D**). The localization of CNVs in the *in silico* chromosomes and hybridization of chromosomal bands with chromosome-specific markers are shown at the top and bottom in each panel, respectively. **Figure 4A** shows the rearrangement identified with two specific markers from chromosome TcChr8 that hybridized with a single chromosomal band of 0.59 Mb in the G strain and two bands of 0.57 and 0.71 Mb in clone D11, indicating the presence of two homologous chromosomes of the same size (0.59 Mb) in the parental G strain and two different-sized homologous chromosomes in clone D11. Chromoblot hybridization results suggest that the 0.71 Mb chromosome of clone D11 was the result of an internal duplication of a 120 kb region in one of the homologous of the G strain. However, the duplication was not identified by aCGH in TcChr8 homologues (**Figure 4A**), suggesting that it may have occurred in a region that was not represented in the DNA microarray. For instance, some unresolved gaps located at the subtelomeric and non-syntenic regions of *T. cruzi* genome (El-Sayed et al., 2005; Berná et al., 2018). Only two short chromosomal alterations, a 33.8 kb deletion and a 11.3 kb duplication, were detected by aCGH in the D11 chromosome homologous to TcChr8 (**Figure 4A**).

Taken together, these results suggest the involvement of both homologous chromosomes of the G strain in the chromosomal rearrangement found in clone D11. We also suggest that the 0.71 Mb chromosome of clone D11 was the result of an internal segmental duplication in one of the homologous chromosomes of the G strain while the 0.57 Mb chromosome of D11 was generated by loss of a (20 kb) fragment in the other homologous chromosome. However, we cannot rule out the possibility of translocation of a 120 kb segment from a nonhomologous chromosome to the 0.59 Mb chromosome of the G strain giving rise to the 0.71 Mb chromosome of D11.

**Figure 5:**
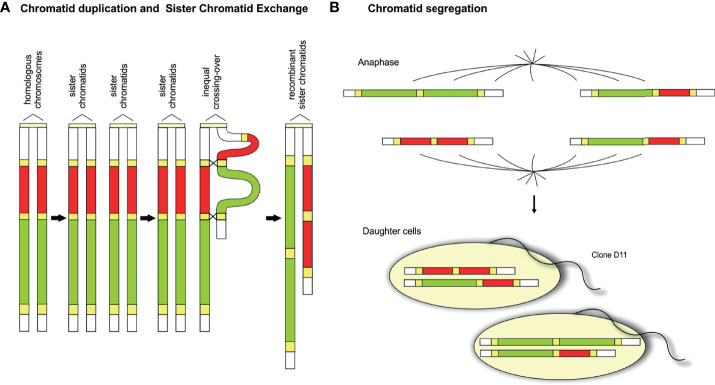
Model of unequal sister chromatid exchange accounting for the rearrangement in the D11 chromosome homologous to TcChr12. **(A)** The pair of parental homologous chromosomes is shown to the left of the duplicated parental sister chromatids. The repeated sequences (pseudogenes RHS, TS and DGF-1) (in yellow) flanking the regions involved in the duplication (red) and deletion (green) recombination events are indicated. After DNA replication, the two sister chromatids are paired with slippage of repeated sequences prior to recombination. Unequal sister chromatid cross over resulting in two chromatids of different sizes. **(B)** At the end of mitotic division two sets of homologous chromosomes are separated from each other in the daughter cells. *Each daughter cell receives* a sister recombinant chromatid. Clone D11 receives the smaller recombinant chromatid resulting from tandem duplication of a segment (in red) and deletion of the adjacent segment (in green).

Hybridization of the TcChr12-specific markers identified a single chromosomal band in both isolates, one band of 0.59 Mb in the G strain and another of 0.64 Mb in clone D11 (**Figures 4B** and **Supplementary Figure S8B**). However, the aCGH analysis showed two chromosomal alterations adjacent to each other, a duplicated and a deleted region of approximately 139 kb and 278 kb, respectively. Gain and loss regions are flanked at both sides by members of MGFs, including pseudogenes of RHS, trans-sialidases and DGF, allowing misalignment between sister or homologous chromatids. The rearrangement may be explained by unequal sister chromatid exchange that resulted in deletion and duplication of chromosome segments in the same chromatid. As shown in **Figure 5**, an unequal sister chromatid crossing over resulted in two different-sized sister chromatids (**Figure 5A**). The smaller sister chromatid lost an ~250 kb segment (in green) while it gained a segment of ~140 kb, resulting in a duplication of ~280 kb (in red). In the meantime, the larger sister chromatid lost the 140 kb segment and gained 250 kb (**Figure 5A**). At the end of mitotic division there were two sets of homologous chromosomes in the daughter cells (**Figure 5B**). In clone D11, one homologous chromosome was represented by the smaller sister chromatid whereas the other homologous chromosome was similar to the parental chromatid. The use of aCGH allowed the identification of cryptic imbalances, which had not been detected by chromoblot hybridization.

Some considerations need to be made when comparing the size of an in silico chromosome with that of the chromosomal band separated by PFGE. The current sequenced genomes of *T. cruzi* are more complete and precise. However, a very few chromosomes have been finished end to end (telomere to telomere), and some unresolved gaps persist. In this way, in silico chromosomes can be smaller than the chromosomal bands in which they were assigned. The models proposed to explain the chromosomal rearrangements are based on the size of in silico chromosomes that makes difficult to correlate accurately the size of aCGH alterations with the size of chromosomal band. Regarding the chromosome TcChr6, the deletion identified by aCGH in one of the chromatids could have been balanced by DNA gain of the same size that occurred in the gaps or subtelomeric regions that are not represented in the DNA array. The presence of a single chromosomal band in clone D11 instead of two bands, as expected by the proposed model (**Figure 5**), could be explained by DNA gain occurring in the gaps and/or subtelomeric regions. In a previous work, Lima et al., 2013 (Lima et al., 2013) demonstrated that telomeric regions were involved in the sized-chromosome polymorphism in clone D11. These regions are very polymorphic and for this reason they were not included in the DNA array.

**Figure 4C** shows the rearrangement found in the D11 chromosome homologous to TcChr6. A specific-chromosome marker of TcChr6 hybridized with two chromosomal bands of 0.73 and 0.68 Mb in the G strain and with a single smaller-sized band of 0.64 Mb in clone D11, indicating the presence of two different-sized homologous chromosomes in the parental strain. Regarding the clone D11, the size of one of the homologous chromosomes can be estimated at 0.64 Mb while the other homologous chromosome would have a smaller size. Chromoblot hybridization corroborated the aCGH finding of a large deletion in TcChr6 (-1 log2 ratio). This rearrangement may be explained by unequal crossing over between homologous chromatids with deletion of an ~280 kb segment resulting in two different-sized DNA molecules (**Supplementary Figure S9A**). After chromosome segregation, clone D11 receives one recombinant homologous chromosome represented by the smaller chromatid, and the other homologous chromosome is similar to the parental chromatid (**Supplementary Figure S9B**).

TcChr22-chromosome markers hybridized with two chromosomal bands of the parental G strain (1.29 Mb and 0.96 Mb) but with only one band in clone D11 (1.17 Mb) (**Figure 4D**). The assignment of TcChr22 markers into two bands in the G strain indicated the presence of a pair of different-sized homologous chromosomes. The chromosome size differences between clone D11 and the parental strain were 0.12 Mb and 0.21 Mb, suggesting small chromosomal rearrangements. After analyzing the aCGH results, we were able to detect a 148 kb deletion in one of the haplotypes of clone D11. The hybridization of the marker TcCLB.509499.20 to the 1.17 Mb chromosomal band supports that the 148 kb segment identified by aCGH is present in one homologous chromosome of clone D11 (**Figure 4D**). One of the homologous chromosomes of clone D11 may have resulted from a deletion in the 1.29 Mb chromosome of the G strain while the other may have arisen from a segmental duplication in the 0.96 Mb chromosome of the G strain.

**Figure 6:**
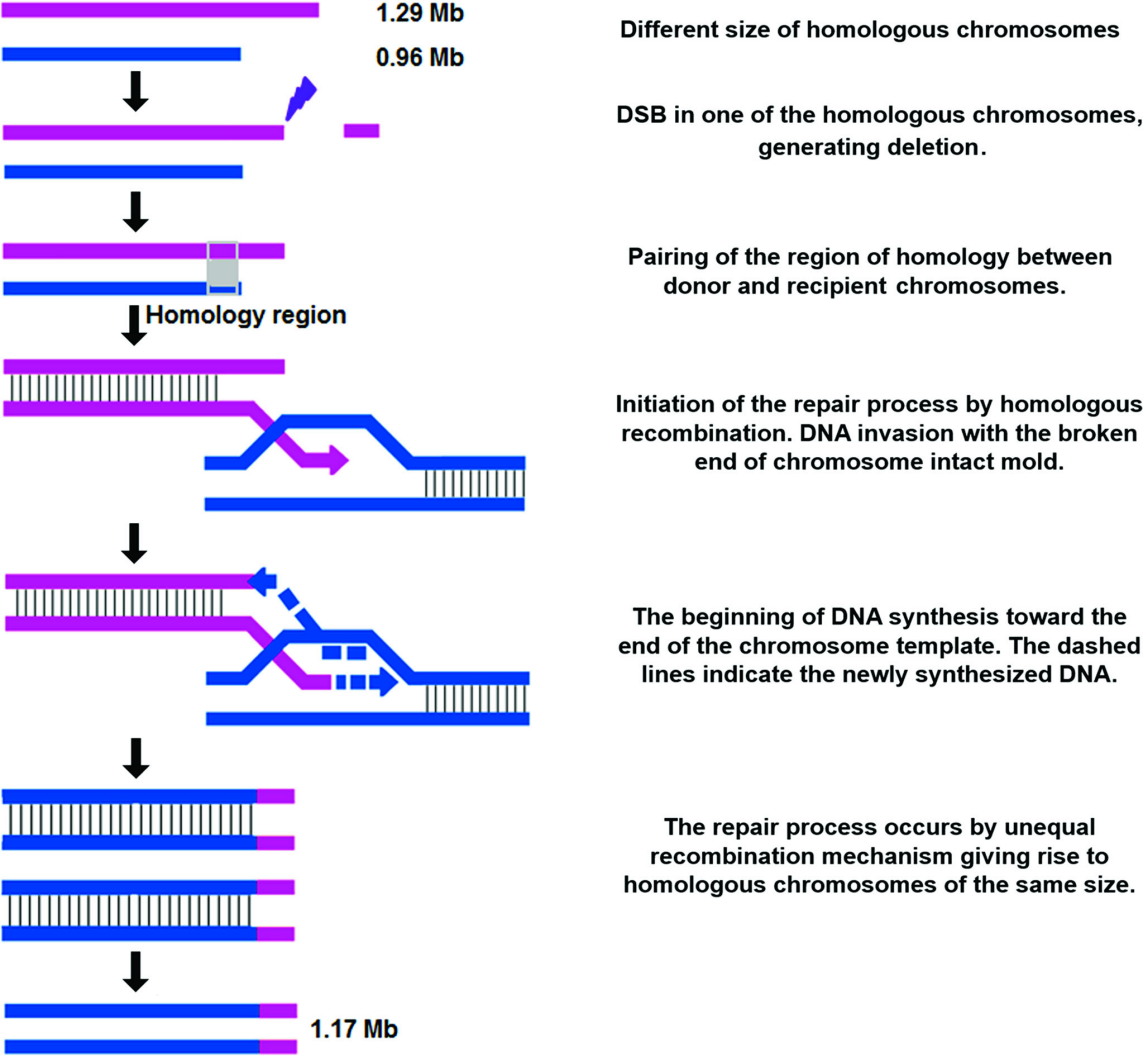
Unequal homologous recombination *via* break-induced replication (BIR). Possible mechanism of homologous recombination that gives rise to TcChr 22 polymorphism in D11 clone.

Although the chromosomal rearrangement in the D11 chromosome homologous to TcChr22 is quite complex, it allows a comprehensive analysis of the molecular mechanisms involved. The TcChr22-specific markers were mapped to two chromosomal bands of 1.29 and 0.96 Mb in the G strain and to a single band of 1.17 Mb in the clone D11. Lima et al., 2013 (Lima et al., 2013) suggested that fusion of the different-sized homologous chromosomes of 1.29 and 0.94 Mb of the G strain generating a dicentric chromosome was followed by fission, resulting in two similar-sized homologous chromosomes (1.17 Mb) in clone D11. However, the aCGH analysis showed a 148 kb deletion in one homologous chromosome of clone D11. Since the recombination model proposed by Lima et al., 2013 (Lima et al., 2013) did not foresee a DNA loss, we decided to propose a new model (**Figure 6**), according to which during the cloning process a double-strand break (DSB) occurred in the 1.29 Mb homologous chromosome of the G strain. The break occurred in a region that did not share any homology with the 0.94 Mb homologous chromosome of the G strain. The pairing of homologous chromosomal regions was followed by DNA repair through unequal homologous recombination (HR) *via* break-induced replication (BIR). First, the chromosomal extremity with the DSB recombination annealed with a homologous region of the DNA donor, initiating the replication repair. At the DSB end the strand resection occurred while the other strain invaded the homologous sequence in the intact donor DNA. The repair mechanism generated two homologous chromosomes measuring 1.17 Mb as a result of the addition of a 110 kb region to the donor chromosome.

### aCGH Data Validation by qPCR

Quantitative real time PCR (qPCR) was used to validate chromosomal alterations that represent different predicted copy number variations (loss, gain or null) in clone D11 (**Table 1** and **Supplementary Table S4**). Triplicate qPCR assays were performed, and the results showed that 7 out of 9 aCGH results (77.7%) were confirmed (**Table 1** and **Supplementary Figure S10**). Two alterations not validated by qPCR correspond to DNA loss and were covered by a large number of probes in the aCGH. These data lead us to suggest that the outcome of aCGH should be considered correct even when it cannot be validated by qPCR.

**Table 1:** Results of quantitative real time PCR analysis for aCGH validation.

TcChr	Alteration			Type*	Validated	Genes		
	Start	End	Frequency			Gene ID	Start	End
6	128524	348838	0,942	loss	no	TcCLB.507603.180	302856	306257
8**			0,026	–	yes	TcCLB.510337.30	146763	148151
12	9715	234335	0,335	gain	yes	TcCLB.503595.10	123806	125734
12	251096	476676	0,528	loss	yes	TcCLB.509573.40	439833	440546
13***	11738	22832	0,202	–	yes	TcCLB.511815.90	200717	202384
13***	379181	475473	0,141	–	yes	TcCLB.511827.50	465082	466506
22	82493	231475	0,311	loss	no	TcCLB.509499.20	118952	120400
22**			0,007	–	yes	TcCLB.504427.170	310344	311150
24	242813	444364	0,203	loss	yes	TcCLB.509123.30	248254	248985

*The minus sign (-) indicates no alteration between parental strain G and clone D11.

**Chromosome region without copy number changes detected by aCGH.

***Chromosome region with copy number changes detected by aCGH.

In our work, the CNV validation rate when we used qPCR was similar to that obtained in mammalian genomes (Li et al., 2012; Wang et al., 2013; Wang et al., 2015; Baldan et al., 2019) observed that the qPCR validation rate is directly related to the number of probes in the region where aCGH detected alterations. They suggested that validation is necessary when the alteration is detected by fewer than 10 probes. Here, two alterations detected by aCGH were not confirmed by qPCR (**Table 1**) but were confirmed by a large number of probes, 257 on chromosome TcChr 22 (TcCLB.509499.20) and 149 on chromosome TcChr 6 (TcCLB.507603.180) (**Supplementary Table S2**). Furthermore, some sequences are known not to amplify in the PCR reaction because Taq polymerase lacks 3’→5’ exonuclease activity (Tindall and Kunkel, 1988; Wu et al., 1989). The presence of a mismatch in the last nucleotide at the 3’ end of the primer is enough to block the extension of the oligonucleotide by Taq DNA polymerase as there is a lack of 3’→5’ exonuclease activity (Wu et al., 1989) It is possible that because of the large size of the probes in aCGH, the chances of finding SNP-type polymorphisms using this technique are higher. This would explain why aCGH is able to detect variants that cannot be detected (amplified) by PCR.

## Discussion

Clone D11 is a derived clone of the *T. cruzi* G strain isolated in our laboratory by a limiting dilution method (Santori, 1991; Lima et al., 2013). The parental G strain was isolated in 1980 from an opossum (*Didelphis marsupialis*) in the Brazilian Amazon (Yoshida, 1983; Yoshida, 2006). Over the years the G strain has shown consistent phenotypic stability for virulence, growth and metacyclogenesis. For instance, the infective metacyclic trypomastigotes display the same profile of cell surface glycoproteins, as well as the same ability to invade human epithelial cells, which is associated with the expression of cell surface glycoproteins and differential calcium signaling activity (Teixeira and Yoshida, 1986; Mortara et al., 1988; Schenkman et al., 1988; Yoshida et al., 1989; Mortara et al., 1992; Ruiz et al., 1998; Yoshida, 2006; Atayde et al., 2007). Genotyping analysis using 10 different microsatellite loci suggested that the G strain displays a monoclonal population structure (Lima et al., 2013). Clone D11 differs from the parental G strain in five microsatellite loci, suggesting that it might have been generated during the cloning process rather than isolated from a pre-existing mixed population (Lima et al., 2013). On the other hand, we cannot rule out the hypothesis the existence of a multiclonal population structure formed by subpopulations with some differences from the original predominant strain. Taking into account these possibilities, we have assumed that clone D11 is derived from G strain. Here, we designed a high-resolution array based on CLB *in silico* chromosome sequences using the e-array platform from Agilent technologies, Santa Clara, California, USA. The aCGH analysis was performed to compare the chromosomal alterations in the G strain and clone D11, and the CLB genome was used as a reference. The aCGH analysis performed by Minning et al. (2011) highlighted the gene content variability among *T. cruzi* isolates, elucidating the impressive interstrain variation in this parasite. In the present study, we showed intrastrain genetic variability between a clone and its parental strain. We identified a high number of small DNA deletions in clone D11, a finding which is in agreement with previous estimates that the D11 genome (81 Mb) is smaller than the G strain genome (89.8 Mb) (Lima et al., 2013).

Chromosomal alterations (DNA gain or loss) larger than 50 bp between two individuals of the same species are considered the main source of genomic variation (Wang et al., 2015) and are also considered to be an important sources of genetic variation in evolution (Hastings et al., 2009; Anand et al., 2013). Homologous recombination also play a key role to generate genetic exchange and environmental fitness in different organisms and several reports demonstrates homologous recombination also have an important role in *T. cruzi* genetic variability (Chiurillo et al., 2016; Alves et al., 2018). In trypanosomatids, it has been shown that these variations can be due to CNVs that may play an important role in environmental adaptive response and transcript abundance (De Gaudenzi et al., 2011; Downing et al., 2011; Minning et al., 2011; Sterkers et al., 2012; Real et al., 2013; Reis-Cunha et al., 2015; Belew et al., 2017; Dumetz et al., 2017; Reis-Cunha et al., 2018). Therefore, CNVs can affect not only genomic organization but also gene expression (De Gaudenzi et al., 2011; Reis-Cunha et al., 2015; Belew et al., 2017; Dumetz et al., 2017). Several phenotypic and genotypic differences have been detected between clone D11 and the parental G strain (Mortara, 1991; Santori, 1991; Mortara et al., 1999; Mortara et al., 2005; Lima et al., 2013). For instance, extracellular amastigotes of clone D11 were 10–15% less infective to HeLa cells than those of the G strain (Mortara, 1991; Santori, 1991; Mortara et al., 1999). Although chromosome alterations do not seem to affect the fitness of clone D11, some losses could lead to decreasing virulence, suggesting an adaptive role of chromosome instability. Recently, Dumetz et al. (2017)sequenced the genomes and transcriptomes of *Leishmania donovani* in *in vivo* conditions mimicking the natural vector environment and vertebrate host environment. After passage through the insect vector, karyotype changes and CNVs were detected and correlated with the corresponding transcript levels, confirming the impact of aneuploidy on molecular adaptations and cellular fitness. In *T. cruzi*, Belew et al. (2017) performed a comparative transcriptome analysis of clones CLB and CL-14, both derived from the same parental CL strain of *T. cruzi*. Both clones showed reduced or delayed expression of surface antigen genes, which in clone CL-14 was associated with virulence, as this clone is neither infective nor pathogenic in a murine model.

The identification of chromosomes with large chromosomal alterations (≤ 400 kb) in clone D11 suggests the occurrence of segmental aneuploidy. Although aneuploidy is often considered to be deleterious, some fungi and protozoa seem to benefit from it (Pagès et al., 1989; Sterkers et al., 2011; Sterkers et al., 2012; Hirakawa et al., 2015; Reis-Cunha et al., 2015; Möller et al., 2018; Reis-Cunha et al., 2018). *T. cruzi* is considered to be mainly a diploid organism with different-sized homologous chromosomes, but recent studies have suggested the occurrence of aneuploidy in this parasite (Minning et al., 2011; Reis-Cunha et al., 2015; Reis-Cunha et al., 2018). Our data also suggest that segmental aneuploidy is relatively common in *T. cruzi* and could generate genetic diversity in an organism in which reproduction seems to be predominantly asexual.

The *T. cruzi* genome contains a large number of repetitive sequences (micro and minisatellites, retrotransposons and MGFs) (El-Sayed et al., 2005; Berná et al., 2018; Callejas-Hernández et al., 2018). It has been proposed that the genome is structured in a “core compartment” composed of conserved genes and conserved hypothetical genes, and a nonsyntenic region (“disruptive compartment”) composed of the multigene families TS, MASP and mucins (Berná et al., 2018). Several MGFs (GP63, DGF-1 and RHS) are dispersed throughout both compartments (Berná et al., 2018). These regions of *T. cruzi* rich in repetitive sequences (“disruptive compartments”) could serve as recombination sites for homologous recombination (El-Sayed et al., 2005; Kim et al., 2005; Martins et al., 2008; Bartholomeu et al., 2009; Moraes Barros et al., 2012; Chiurillo et al., 2016; Talavera-López et al., 2021). Comparison of the gene annotation in altered regions of clone D11 with that of the corresponding regions in CLB indicated an increase in MGFs. When we compared the chromosomal alterations with the gene annotation, we could observe members of MGFs flanking deletions and amplifications in the D11 clone, suggesting that these genes play a role in recombination events. There was an increase in MASP genes and mucin and gp63 in the amplification regions in the D11 clone while in the deletion regions there was a reduction in these genes and an increase in RHS and DGF-1. These results suggest that MGFs may have a role in the homologous recombination, serving as homology site.

Gross chromosome rearrangements in clone D11 were detected by chromoblot hybridization and supported by aCGH. We suggest that mitotic chromosome rearrangements may be explained at molecular and cytological levels by unequal crossing over between sister or homologous chromatids mediated by flanking repeated sequences and unequal homologous recombination *via* break-induced replication. For instance, the rearrangements of chromosomes TcChr12 and TcChr6 may be generated by unequal mitotic crossing over between sister or homologous chromatids which are justified by the rearrangements in chromosomes TcChr12 and TcChr6, respectively. Another mechanism is the unequal HR *via* BIR proposed to explain the TCcr22 rearrangement. Homologous recombination has been demonstrated in *T. cruzi* natural populations and in experimental conditions, in which it mediates the integration of exogenous DNA from the transfection vector into the trypanosome genome (Gaunt et al., 2003; Baptista et al., 2004; Passos-Silva et al., 2010; Ramírez et al., 2013; Chiurillo et al., 2016). One of the best-studied recombination mechanisms is that induced by DNA DSBs (Hastings et al., 2009; Sakofsky et al., 2012; Anand et al., 2013). A DSB is a potentially lethal event that can occur by exposure to ionizing radiation and chemical mutagens, but can also occur spontaneously during DNA replication and segregation (Hastings et al., 2009; Sakofsky et al., 2012; Anand et al., 2013). HR involves the interaction between large homology regions and single strand annealing (SSA), gene conversion or BIR (Hastings et al., 2009; Sakofsky et al., 2012; Anand et al., 2013). The repair occurs by BIR when only one end of a DSB has homology with the donor sequence; one strand is resected, and the other invades the homologous sequence in the intact donor DNA to initiate the repair. BIR repair results in a copy of several kilobases from the donor site and DNA loss.

Accumulating experimental evidence suggests that recombination has driven major genetic alterations in *T. cruzi* (Tibayrenc et al., 1990; Westenberger et al., 2005; de Freitas et al., 2006; Tibayrenc and Ayala, 2013; Berry et al., 2019; Schwabl et al., 2019). Recently, it has been suggested that homologous recombination may play an important role in the dormancy signaling in *T. cruzi*. Sánchez-Valdéz et al. (2018) (Sánchez-Valdéz et al., 2018) described the occurrence of spontaneous dormancy in the amastigote during extended drug exposure in the experimental *T. cruzi* infection. These authors hypothesized that dormancy could be caused by genetic recombination events in the replicating stages of *T. cruzi* life cycle (Sánchez-Valdéz et al., 2018). Resende et al. (2020) (Resende et al., 2020) demonstrated that homologous recombination plays an important role in the dormancy in *T. cruzi*. They showed that amastigote and epimastigote dormancy is strain-dependent in *T. cruzi* and it is directly correlated with mRNA TcRAD51 levels, a recombination pivot, responsible for strand invasion and search for homology between broken strand and the template (Baumann and West, 1998; Gomes Passos Silva et al., 2018; Resende et al., 2020). Costa-Silva et al. (2021) demonstrated that DNA topoisomerase 3α (TcTopo3α) is important for homologous recombination repair and replication stress in *T. cruzi*. TcTopo3α gene knockout inhibited the amastigote proliferation and a high number of dormant cells was identified. TcTopo3α knockout parasites treated with methyl methanesulfonate (MMS) showed a slower cell growth and loss their ability to repair damage. Segmental duplication/deletions were associated to the multigene family’s regions in the knockout parasites (Costa-Silva et al., 2021).

The genomic changes detected by aCGH suggest the presence of a dynamic genome that responds to environmental stress by varying the number of gene copies and generating segmental aneuploidy.

## Data Availability Statement

The original contributions presented in the study are included in the article/**Supplementary Material**. Further inquiries can be directed to the corresponding authors. The raw aCGH data and sequence of probes involved in the aCGH design for Trypanosoma cruzi have been deposited into the GenBank GEO database (GSE197870).

## Author Contributions

Conceived and designed the experiments: MM, JS, DB, and SR. Performed the experiments: DC, MM, FL, JR-C, RC, FM, RV, and AC-M. Analyzed and interpreted the data: MM, DC, and JS. MM and JS wrote the paper with contributions from all authors. All authors read and approved the final manuscript.

## Funding

This work was supported by the thematic project FAPESP (2016/15000-04) coordinated by Renato Arruda Mortara (RAM), and in this project José Franco da Silveira (JS) participates as deputy coordinator. As recommended by FAPESP, the name of the coordinator must appear together with the process number in the Funding section, as follows: Renato Arruda Mortara (2016/15000-04). AC-M is a postdoctoral fellowship from CAPES/PNPD, Brazil (Coordenação de Aperfeiçoamento de Pessoal de Nível Superior/Programa Nacional de Pós-Doutorado). This study was also supported by Conselho Nacional de Desenvolvimento Científico e Tecnológico (CNPq), Brazil, as follows: doctoral fellowship for RC (147453/2016-0), postdoctoral fellowship for MM (157637/2015-8) and DC (150526/2017-2) and PQ for JS (306591/2015-4) and DB (309465/2015-0); and in part by CAPES and the Instituto Nacional de Ciência e Tecnologia de Estratégias de Interação Patógeno-Hospedeiro INCT-IPH), CNPq, Fundação à Pesquisa do Estado de Goiás (FAPEG), Brazil.

## Acknowledgments

We would like to thank Dr Yuri Moreira (Agilent Brasil) for his expert advice and technical support for the T. cruzi-CGH array, and to Drs Thaise L. Teixeira and Camila M. Yonamine Asanuma for the advice and support in the preparation of the manuscript.

## Conflict of Interest

The authors declare that the research was conducted in the absence of any commercial or financial relationships that could be construed as a potential conflict of interest.

## Publisher’s Note

All claims expressed in this article are solely those of the authors and do not necessarily represent those of their affiliated organizations, or those of the publisher, the editors and the reviewers. Any product that may be evaluated in this article, or claim that may be made by its manufacturer, is not guaranteed or endorsed by the publisher.

## Supplementary Material

Supplementary Figure 1 Alignment between homologous regions of T. cruzi in sílico assembled chromosomes of clone CLB (lineage TcVI) and Dm28c (lineage TcI). Pairwise comparisons with ACT (Artemis Comparison Tool) between the chromosomes TcChr4, TcChr6, TcChr8, TcChr12 and TcChr22 (“S” chromosome assigned to the Esmeraldo haplotype and “P” to the non-Esmeraldo haplotype) of the clone CLB and the homologous chromosomes of Dm28c (version 2018). Homologous genes are connected by colored lines. The matches and reverse matches are represented in red and in blue, respectively. Grey blocks represent each chromosome.

Supplementary Figure 2 The size-class distribution of CNVs identified by aCGH in clone D11. Chromosomal alterations (n=418) were identified in clone D11 by comparison to the parental G strain. On the axes Y and X are shown the number and size (kb) of chromosomal alterations, respectively.

Supplementary Figure 3 Proportion of total CNVRs length in each T. cruzi chromosome that is copy number variable in clone D11. The proportion of chromosome-size alterations (loss or gain) in each chromosome of D11 was determined using the in silico chromosomes of CLB as a reference (Weatherly et al., 2009). Chromosomal alterations identified in clone D11 were mapped on both haplotypes (S, Esmeraldo like and P, Non-Esmeraldo like) on each in silico chromosome of CLB reference genome. The in silico chromosomes of CLB are numbered from 1 to 41, from the smallest TcChr1 (0.77 Mb) to the largest TcChr41 (2.37 Mb). DNA losses or gains are denoted by green and red, respectively. Proportion is on the Y axis.

Supplementary Figure 4 Schematic representation of the in silico chromosomes TcChr6, TcChr8, TcChr12 and TcChr22 showing the gene annotation and chromosomal alterations detected by aCGH in clone D11. Annotation of multigene families in CLB is shown in light blue and the other genes in black. The CNVs detected by aCGH in the clone D11 are shown on the in silico CLB chromosomes. DNA amplification and deletion are shown in red and green, respectively. S, Esmeraldo- and P, non-Esmeraldo-like.

Supplementary Figure 5 Gene content in the chromosomal alterations of clone D11. The gene content of amplification and deletion regions of D11 was compared to the gene content of both haplotypes (Esmeraldo and non-Esmeraldo) of the reference genome CLB. The genes annotated in CLB genome were classified into three groups: Hypothetical - genes encoding hypothetical proteins without assigned function; MGF – multigene families encoding surface proteins, such as, trans-sialidases, MASPs, TcMUC, GP63 and DGF1, and nuclear proteins coded by RHS gene family (retrotransposon hot-spot protein); and others – DNA replication genes, kinases, phosphatases, etc. The CLB gene content was compared with those in the amplification and deletion regions of clone D11 (left).

Supplementary Figure 6 Schematic representation of the in silico chromosomes of non-Esmeraldo-like haplotype showing the gene annotation and chromosomal alterations detected by aCGH in clone D11. Annotation of multigene families in CLB is shown in light blue and the other genes in black. The CNVs detected by aCGH in the clone D11 are shown on the in silico CLB chromosomes. DNA amplification and deletion are shown in red and green, respectively. The chromosomes and recombination events are shown to scale (Bar=200kb).

Supplementary Figure 7 Molecular karyotypes of clone D11 and the parental G strain. Panel **(A)** Chromosomal bands were resolved by PFGE and stained with ethidium bromide. The visualized bands were named using capital letters (21 bands, A – U) (Lima et al., 2013) for clone D11 and Arabic numerals (19 bands, 1 -19) (Souza et al., 2011) for G strain. Panel **(B)** Schematic representation of the molecular karyotype of clone D11 and G strain showing the molecular weight of each band on the right side of the strip. The thickness of the rectangles represents the thickness of each visualized chromosomal band. C) Raw image showing the pulsed field electrophoresis running gel. Note: The karyotype of D11 and the G strain had already been described by Lima et al., 2013 (Lima et al., 2013). The electrophoretic runs shown in **Figure S7** were performed in the present work. They have included as a **Supplementary Figure** to facilitate the understanding of mapping of aCGH alterations in the chromosomal bands of the G strain and clone D11.

Supplementary Figure 8 Raw images showing the pulsed field electrophoresis running gels and Southern blot hybridizations from. Upper Panel. Separation of T. cruzi chromosomal bands on a pulsed field gel (PFGE). Chromosomal DNA was prepared from T. cruzi isolates: clone CL Brener (CLB) used as a reference genome in this study, strain G (parental strain) and clone D11, a single-cell derived of G. strain. Hansenula wingei (HW) chromosomes were used as PFGE DNA size markers. The lengths of HW chromosome bands are indicated in megabases (Mb) to the left of the gel. The boxed regions of each gel were used in the Southern blot hybridization. Botton Panel. The chromosomal bands were transferred onto nylon membranes and hybridized to the indicated chromosome markers. The identification (number or letter) and size (Mb) of hybridizing chromosomal bands are indicated in the figure. In silico chromosomes and markers are: **(A)** TcChr8, TcCLB.510337.30 – adenylosuccinate lyase; TcCLB.506649.50 - cytochrome c oxidase assembly factor; **(B)** TcChr12, TcCLB.509573.40 - hypothetical protein**;** TcCLB.503595.10 - hypothetical protein; **(C)** TcChr6; TcCLB.507603.180 - ubiquitin hydrolase 6; **(D)** TcChr22; TcCLB.509499.20 - beta propeller protein 1 BPI; TcCLB.504427.89 - SET domain containing protein; TcCLB.509767.180 - exosome componente CSL4.

Supplementary Figure 9 A model of unequal crossing over accounting for the rearrangement in D11 chromosome homologous to TcChr6. **(A)** First it is shown the pair of parental homologous chromosomes, next the duplicated parental sister chromatids. The repeated sequences (pseudogenes of RHS, TS and DGF-1) (in yellow) flanking the regions involved in the deletion (green) recombination events are indicated. After mitotic DNA replication, the homologous chromatids are paired with slipping of repeated sequences prior to recombination. Unequal homologous chromatid cross-over resulting in two DNA molecules of different sizes. **(B)** At the end of mitotic division two sets of homologous chromosomes are separated from each other in the daughter cells. Each daughter cell received a homologous recombinant chromatid. Clone D11 received the smaller recombinant chromatid generated by deletion of a large segment (in green). S, Esmeraldo haplotype; P, non-Esmeraldo haplotype.

Supplementary Figure 10 Real-time PCR estimation of gene copy number of markers in G strain and clone D11 for aCGH validation. Genes located in different chromosomes were amplified from the genomic DNA of the strain G and clone D11 and detected by SYBR Green. The graphs represent mean ± SD of three independent experiments performed in triplicate. Comparison between G and D11 strain was determined using Unpaired Student t test. P values were shown in the figure.
