# Supplementary figures and images for: Trypanosoma cruzi Genomic Variability: Array Comparative Genomic Hybridization Analysis of Clone and Parental Strain
Source: Front Cell Infect Microbiol. 2022 Mar 25;12:760830. doi: 10.3389/fcimb.2022.760830 (PMC8992781; doi:10.3389/fcimb.2022.760830)

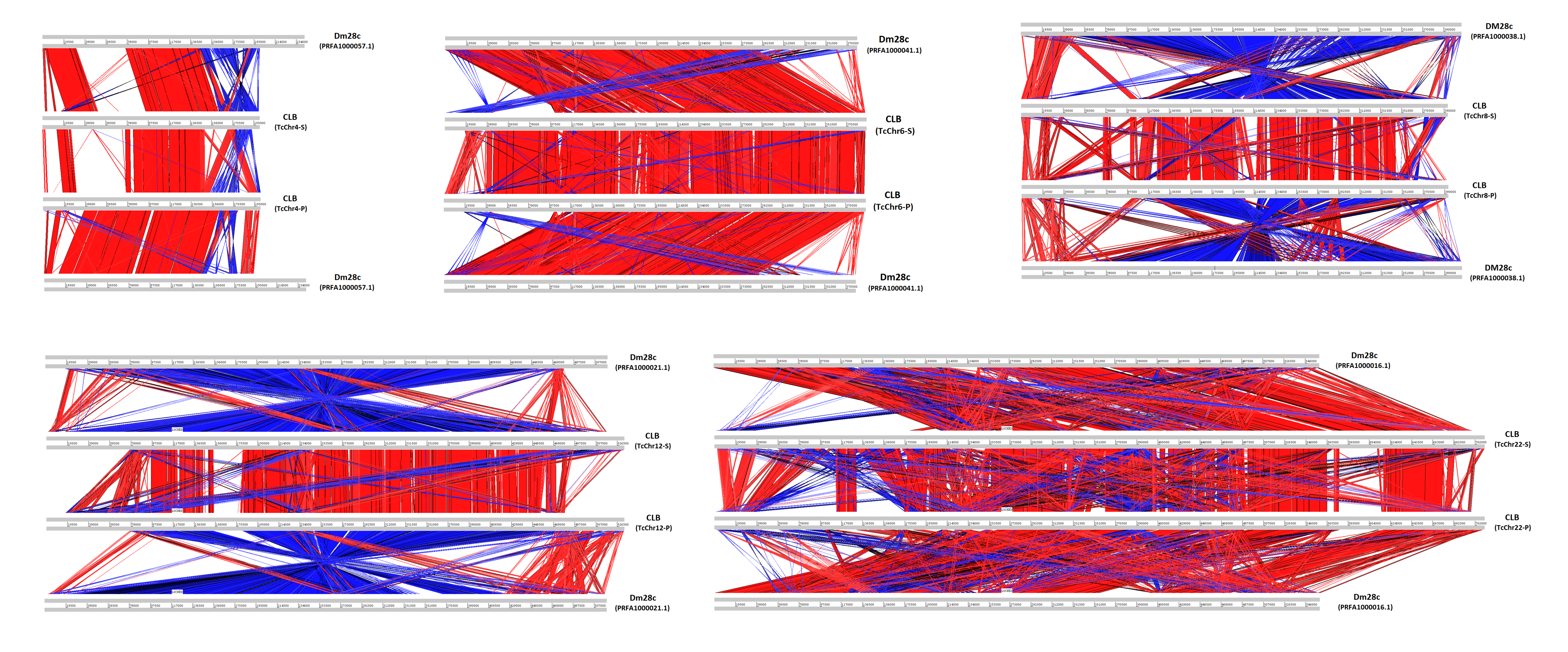

Supplement: Supplementary Figure 1 — Alignment between homologous regions of T. cruzi in sílico assembled chromosomes of clone CLB (lineage TcVI) and Dm28c (lineage TcI). Pairwise comparisons with ACT (Artemis Comparison Tool) between the chromosomes TcChr4, TcChr6, TcChr8, TcChr12 and TcChr22 (“S” chromosome assigned to the Esmeraldo haplotype and “P” to the non-Esmeraldo haplotype) of the clone CLB and the homologous chromosomes of Dm28c (version 2018). Homologous genes are connected by colored lines. The matches and reverse matches are represented in red and in blue, respectively. Grey blocks represent each chromosome. [file Image_1.tif]

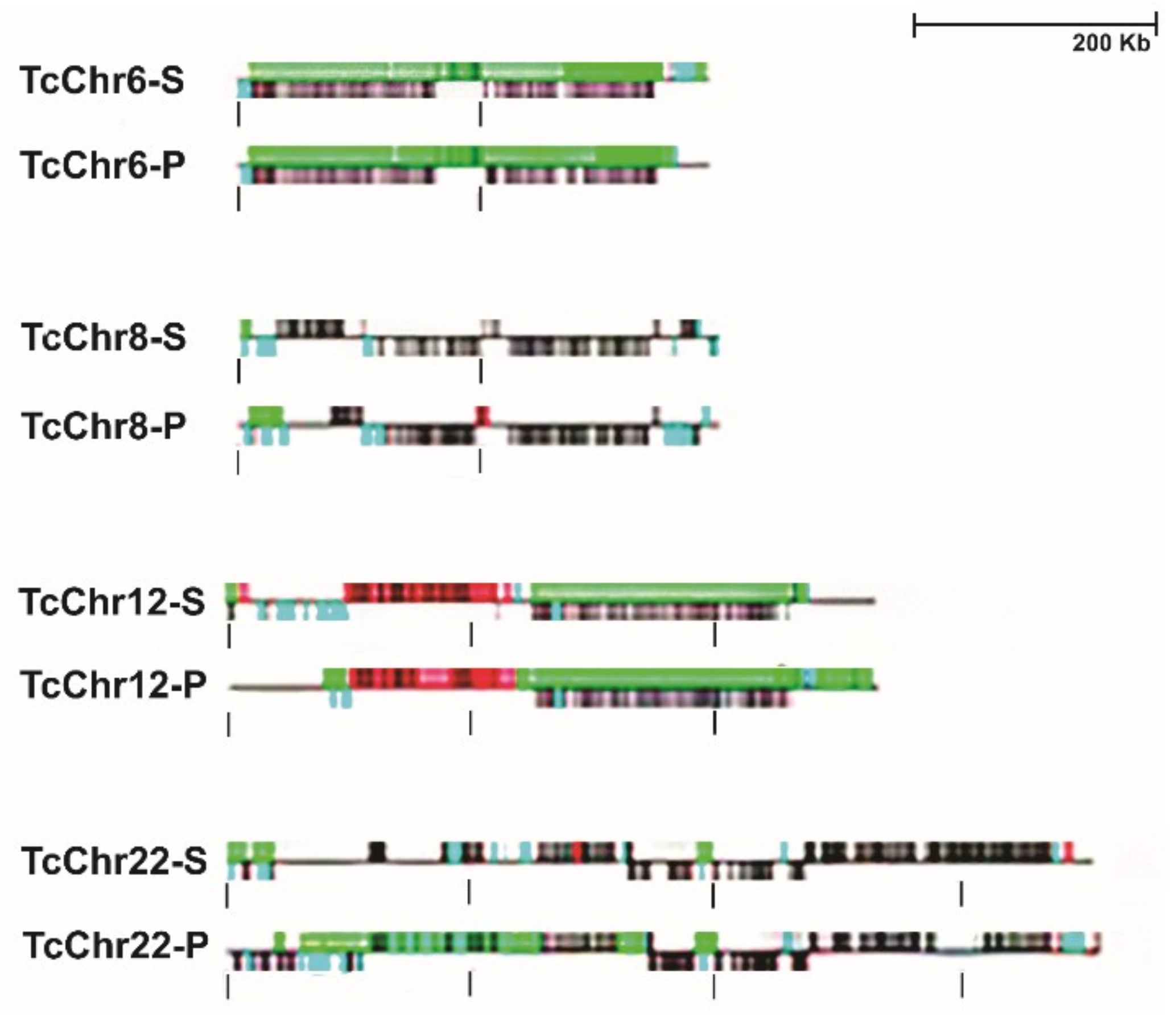

Supplement: Supplementary Figure 4 — Schematic representation of the in silico chromosomes TcChr6, TcChr8, TcChr12 and TcChr22 showing the gene annotation and chromosomal alterations detected by aCGH in clone D11. Annotation of multigene families in CLB is shown in light blue and the other genes in black. The CNVs detected by aCGH in the clone D11 are shown on the in silico CLB chromosomes. DNA amplification and deletion are shown in red and green, respectively. S, Esmeraldo- and P, non-Esmeraldo-like. [file Image_4.tif]

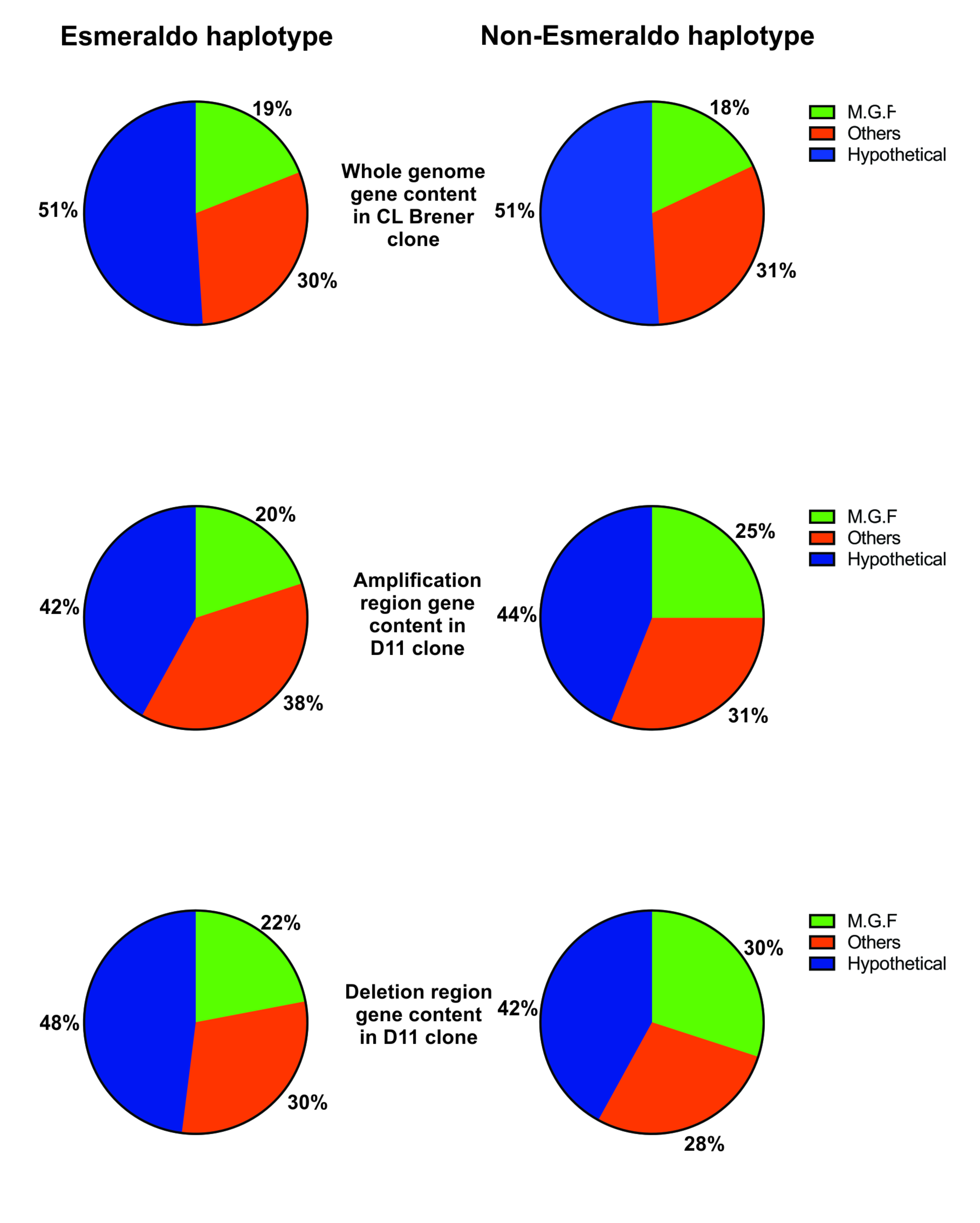

Supplement: Supplementary Figure 5 — Gene content in the chromosomal alterations of clone D11. The gene content of amplification and deletion regions of D11 was compared to the gene content of both haplotypes (Esmeraldo and non-Esmeraldo) of the reference genome CLB. The genes annotated in CLB genome were classified into three groups: Hypothetical - genes encoding hypothetical proteins without assigned function; MGF – multigene families encoding surface proteins, such as, trans-sialidases, MASPs, TcMUC, GP63 and DGF1, and nuclear proteins coded by RHS gene family (retrotransposon hot-spot protein); and others – DNA replication genes, kinases, phosphatases, etc. The CLB gene content was compared with those in the amplification and deletion regions of clone D11 (left). [file Image_5.tif]

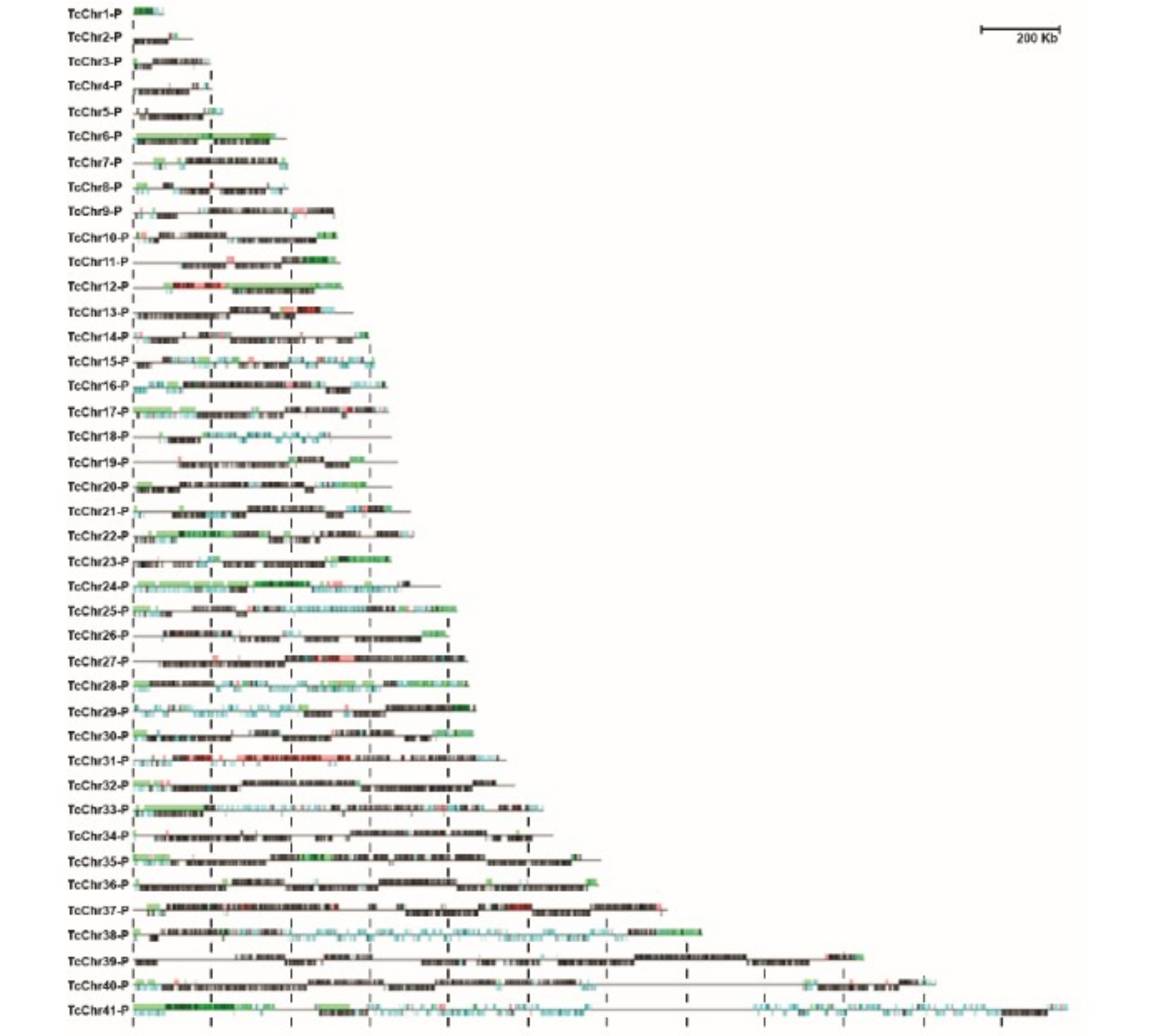

Supplement: Supplementary Figure 6 — Schematic representation of the in silico chromosomes of non-Esmeraldo-like haplotype showing the gene annotation and chromosomal alterations detected by aCGH in clone D11. Annotation of multigene families in CLB is shown in light blue and the other genes in black. The CNVs detected by aCGH in the clone D11 are shown on the in silico CLB chromosomes. DNA amplification and deletion are shown in red and green, respectively. The chromosomes and recombination events are shown to scale (Bar=200kb). [file Image_6.tif]

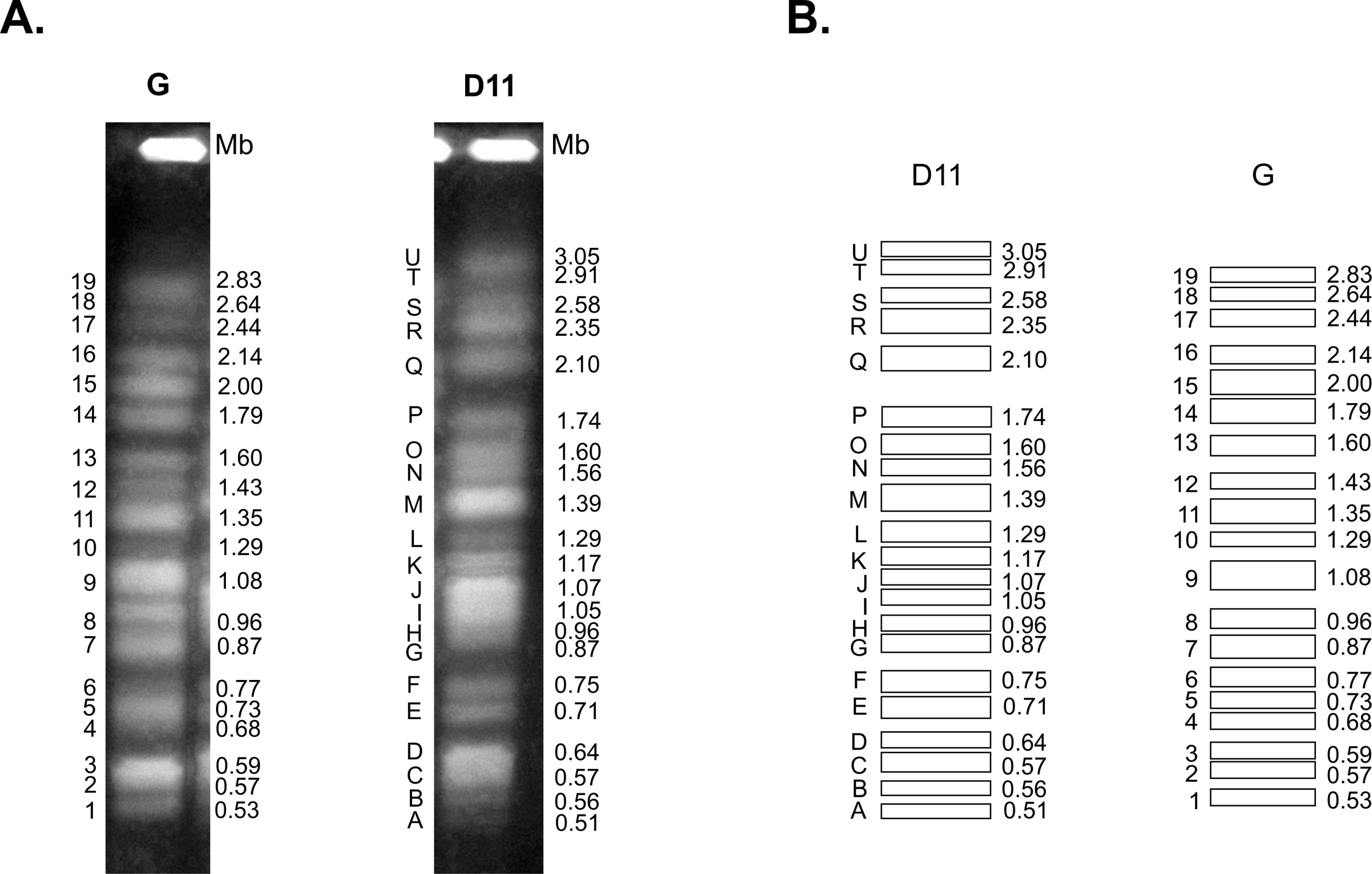

Supplement: Supplementary Figure 7 — Molecular karyotypes of clone D11 and the parental G strain. Panel (A) Chromosomal bands were resolved by PFGE and stained with ethidium bromide. The visualized bands were named using capital letters (21 bands, A – U) (Lima et al., 2013) for clone D11 and Arabic numerals (19 bands, 1 -19) (Souza et al., 2011) for G strain. Panel (B) Schematic representation of the molecular karyotype of clone D11 and G strain showing the molecular weight of each band on the right side of the strip. The thickness of the rectangles represents the thickness of each visualized chromosomal band. C) Raw image showing the pulsed field electrophoresis running gel. Note: The karyotype of D11 and the G strain had already been described by Lima et al., 2013 (Lima et al., 2013). The electrophoretic runs shown in Figure S7 were performed in the present work. They have included as a Supplementary Figure to facilitate the understanding of mapping of aCGH alterations in the chromosomal bands of the G strain and clone D11. [file Image_7.tif]

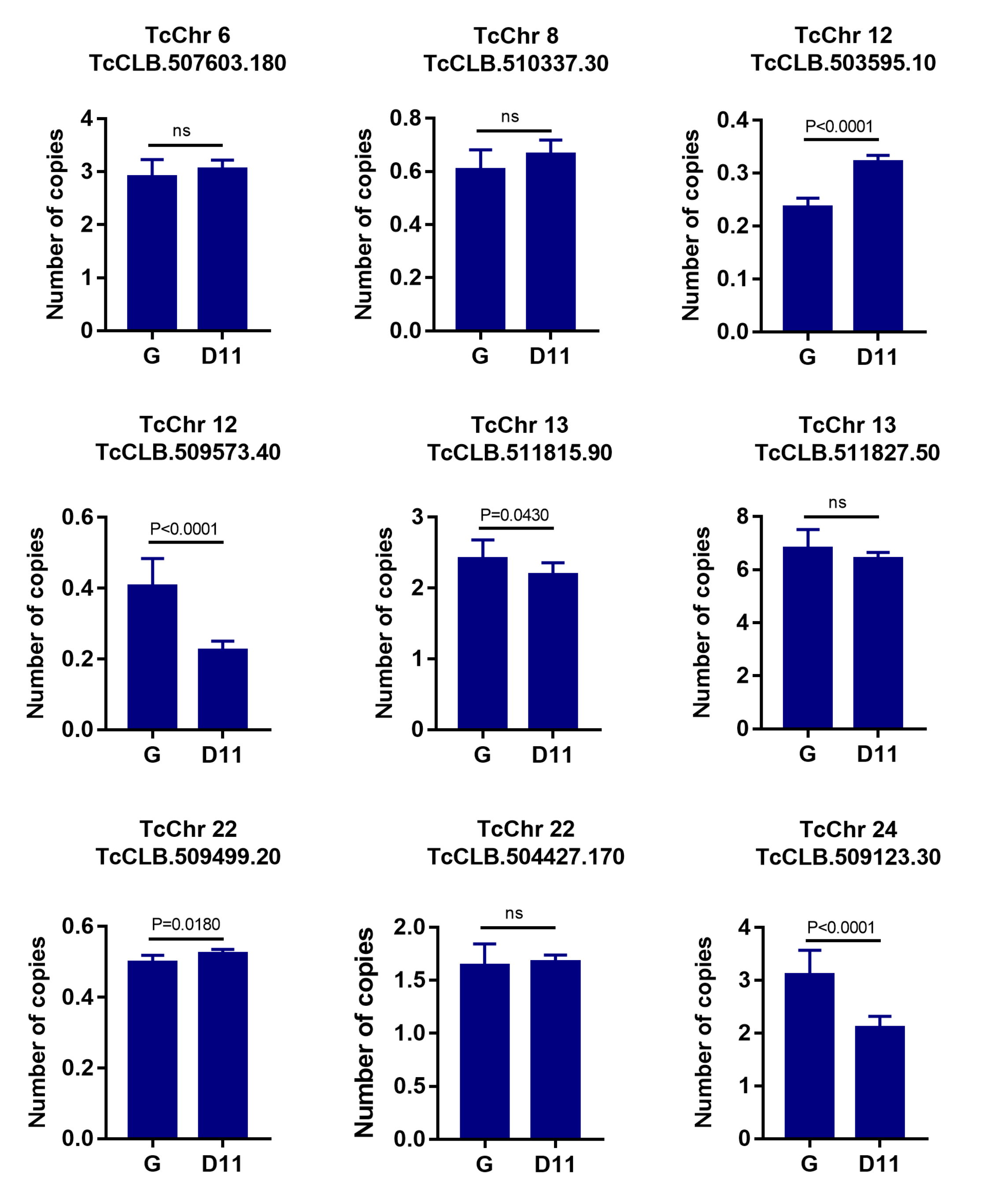

Supplement: Supplementary Figure 10 — Real-time PCR estimation of gene copy number of markers in G strain and clone D11 for aCGH validation. Genes located in different chromosomes were amplified from the genomic DNA of the strain G and clone D11 and detected by SYBR Green. The graphs represent mean ± SD of three independent experiments performed in triplicate. Comparison between G and D11 strain was determined using Unpaired Student t test. P values were shown in the figure. [file Image_10.tif]
